# A neglected critical time to prevent maternal and offspring's adverse outcomes: The preconception period

**DOI:** 10.18502/ijrm.v20i1.10237

**Published:** 2022-02-18

**Authors:** Mojtaba Keikha, Shayesteh Jahanfar, Zeinab Hemati

**Affiliations:** ^1^Department of Public Health, Sirjan School of Medical Sciences, Sirjan, Iran.; ^2^Department of Public Health and Community Medicine, Tufts University School of Medicine, USA.; ^3^Department of Pediatrics and Neonatal Nursing, Child Growth and Development Research Center, Research Institute for Primordial Prevention of Non-Communicable Disease, Isfahan University of Medical Sciences, Isfahan, Iran.

##  Dear Editor, 

At the time of conception, a healthy woman can increase the chances of a successful pregnancy and have a healthy child. But the health of women around the time of conception is an important topic that has been neglected. Now national and international health agencies are paying more attention to this topic. Life-course epidemiology provides a valuable perspective to study the influential factors around conception and potential long-term and latent impacts on the mother's, fetus', and child's health (1). For example, two to three months before and after conception is crucial because of their importance in gamete function and placental development. Therefore, sharper attention to the preconception period can improve mother and child health (2).

Preconception care is a set of interventions to identify and modify women's physical, behavioral, and social risk factors around the conception. In general, preconception care aims to focus on women's health to prevent disease and manage risk factors that affect pregnancy outcomes and the health of future generations. According to the World Health Organization, it is necessary to support all women of reproductive age in preconception care. As the most important period in the development of fetal organs is when many women are unaware of their pregnancy, it is too late for women to contact health care providers to change their lifestyle and receive health promotion advice once they become aware of the pregnancy. There is a growing body of evidence that the preconception lifestyle of men and women can directly impact pregnancy outcomes (3). For example, an unhealthy lifestyle, or being overweight or obese before pregnancy have been recognized as factors associated with weight gain during pregnancy, being overweight after pregnancy and high long-term weight status in both mothers and children. In addition, the preconception period is an excellent opportunity to reduce the risk factors associated with non-communicable diseases in children (4). Epigenetic alterations to gene expression occur soon after conception. Essential factors affecting children's health immediately after conception are obesity, being overweight or malnutrition in the mother, or environmental and social impacts (5).

The Centers for Disease Control and Prevention focus on improvement in meeting men's reproductive health needs. This policy emphasizes the importance of the role of men in the family structure and can lead to paying particular attention to men in preconception care. Men are known to be weak users of preventative health services and are more likely than women to engage in risky health behaviors (6). Health care around preconception provides an opportunity for men to participate in changing their lifestyle, diet and physical activity, and ultimately improve their health, family relationship integrity, and family dynamics. Preconception male well-being takes a comprehensive approach to male health through not isolating their sexual health as a difficulty or disease condition (7).

One of the critical factors that has been recognized as influential in the preconception period is environmental factors, which can affect the health of offspring via maternal and paternal exposures to these environmental factors. Paternal and maternal exposure to environmental pollutants can have adverse consequences, including birth weight reduction due to toxicity and impaired fetal growth. Exposure to chemicals and biological factors, especially persistent organic pollutants, radiation and tobacco smoke, is daily in our lives (8). The impact of these environmental chemicals in the preconception period is through epigenetic changes that occur in the gamete structure in the preconception period and lead to adverse effects on fetal development and offspring health (9).

Numerous medical, behavioral, psychological, and environmental factors are associated with adverse pregnancy outcomes. The most important way to reduce the impact of these factors is to identify and perform appropriate care interventions to correct them. The preconception period is a golden time to prevent the effect of these factors. However, health care alone cannot improve women's health in the preconception period and its impact on fertility outcomes. Therefore, health promotion programs are recommended to change the knowledge and attitudes of women with high-risk behaviors related to fertility, and to develop care programs for couples of reproductive age, to thereby promote the birth of a healthy generation. A healthy fertility program can help women and men of fertile age choose pregnancies based on their goals and values in life. Such programs might increase the proportion of planned pregnancies and encourage women and men to reduce high-risk behaviors before conception and so decrease the risk for offspring's adverse outcomes (10).

Some recommendations are suggested for preconception care to improve maternal and child health:

• To promote preconception health, it is necessary to modify the knowledge, attitudes, and behaviors related to the reproductive health of both men and women.

• Social media and the broader internet can be used to provide information on the environmental, behavioral, and social risks before fertilization that can affect maternal and infant health.

• It is necessary to consider preconception care as an important part of primary and preventative health care. Moreover, monthly visits by health care providers are recommended before conception.

• Almost half of all pregnancies are unwanted; infant low birth weight, maternal postpartum depression, and delay in receiving prenatal care are considered as problems related to this type of pregnancy.

• It is important to prevent pregnancy in adolescence.

• Complicated and long-term health consequences of untreated sexually transmitted diseases might occur in adolescent girls and women. According to the Centers for Disease Control and Prevention, undiagnosed and uncontrolled sexually transmitted diseases could result in infertility in 24,000 females in the United States each yr.

These recommendations are practical for policymakers, public health professionals, clinical care providers, as well as researchers, consumers, and everybody who is concerned with women, children and families.

##  Conflict of Interest

The authors declare that they have no conflict of interest.
